# Meeting of Dec. 19, 1881

**Published:** 1882-01

**Authors:** 


### ARTICLE XII.

CHICAGO MEDICAL SOCIETY. Stated Meeting, December 19, 1881.

Dr. G. C. Paoli occupied the chair, while President E. Ingals, read the paper of the evening on

### THE MANAGEMENT OF PARTURITION.

The writer thought that we would gain as a profession if, instead of so generally directing our investigations into the unexplored and unknown, we would sometimes retrace our steps, and reconsider and reappropriate practices that have been discarded or forgotten.

No class of patients merit our sympathy more than women when in the pangs and perils of labor, and to none do the arts of our profession bring more substantial benefits. Neither physicians nor laymen fully realize the degree of skill which is habitually exercised by the practitioners of obstetrics, or the benefits they confer on those who receive their professional ministrations. Every obstetrician is often obliged to perform operations that require more knowledge, judgment, courage and skill, than do the acts of the surgeon; and they are hardly spoken of beyond the room in which the service is rendered, and should the obstetrician ask such a fee as the surgeon would exact for less difficult operations, the demand would be thought exorbitant. This is because the occasions for obstetric operations are so common, and they are skilfully performed by so many. Considering the skill required, the great responsibility assumed, the unseasonable hours and exhausting labors which the practice of obstetrics involves, the pecuniary returns are more inadequate in this than in any other branch of the profession.

*The Lying-in Chamber.*—The lying-in chamber should be—like every sick-chamber—retired, spacious, pleasant, and capable of being thoroughly ventilated without drafts. The invalid of a house should always be entitled to as good a room as there is in it. An open grate, and a fire in it when the weather will permit, is especially desirable. This matter of the healthfulness of the sick-room is of vastly more importance than is realized. It would overcome the vitality of the most robust person to lie down for a month in a small, closed room, in cold weather heated by a stove, constantly occupied by mother, child and nurse, with floral offerings on the stand, and in which many visits of congratulation are received. The depressing effects of this atmosphere are obvious enough to the physician in the few moments of his professional call, but the patient, becoming habituated to it, is unconscious how impure the air is that she breathes; and wonders at her lassitude, and that she gets up so slowly. But few persons should be admitted to the chamber. During the labor the physician, nurse, and one other female attendant are better than more, and the absence of the husband is generally desirable.

During the first two weeks the patient should be kept excluded from callers; only a very few of her most intimate and judicious friends being allowed to see her for brief periods and at long intervals. We will suppose the patient is seen at the commencement of labor. The manner of the medical attendant in the sick-room is important. He should be quiet and cheerful, and so conduct himself as to impress on the patient the conviction that he feels a genuine interest in her behalf, and that he is competent to conduct the case to a successful issue. I have seen attendants on these occasions who were brusque and rude, with veterinary airs quite unsuited to the place. It is a great point gained when an early examination enables the physician to assure his patient that the presentation is favorable, and that the case may certainly be expected to reach a safe termination. If, however, anything is unpromising, it is best that the patient should be apprised of it; the information being accompanied with the assurance that the difficulty is not beyond the reach of obstetric skill, though it may cause delay and difficulty. This will increase the confidence of the patient in the candor and knowledge of her physician, and will cause her to endure whatever she may be called to bear with increased courage and patience.

If the patient is a primipara, it is better to tell her that she has before her a painful ordeal, that will demand all her fortitude; but that it is only what all mothers have met and passed, and that her rewards will abundantly repay her sufferings. During the first stage of labor the patient may lie down, sit up, or walk about the room, as suits her comfort and inclination, but it is best she should not take too early to the bed. If the lower bowel is not empty, it should be relieved by an enema. When the womb is only commencing to dilate, it is not necessary that the attendant should remain in the house, though he should see the patient often enough to note the progress made, and to relieve her mind from unnecessary anxiety, an examination being made every one or two hours, as the case seems to demand.

The position of the patient during labor is not essential, though most patients prefer the back, and I think it is best, though this may sometimes be advantageously alternated with that of the left side after the labor is well advanced. The placenta is best delivered when the patient is on the back, as in this position the womb can be more effectually manipulated. Should there be considerable pain for a number of hours and no consequent dilatation of the os uteri, morphia should be administered, for such pains are worse than useless. They increase the irritability o the womb, and the organ is contused and injured by its own unavailing contractions. The anodyne will allay the irritabi [illegible]

and promote the dilatation of the os. Patients often attempt to hasten delivery by voluntary expulsive efforts of the abdominal muscles. This both retards the labor and exhausts the strength of the patient, and if permitted at all it should only be when the labor is nearly completed. Such voluntary efforts, however, render valuable aid in the expulsion of the placenta.

During the progress of dilatation all manipulation of the os is injurious. If the labor goes on favorably its progress should be mainly trusted to the spontaneous influences of nature's processes. On this point, however, I do not wish to be misunderstood, for I do not fully endorse the oft-repeated maxim, that "meddlesome midwifery is bad"—for non-meddlesome midwifery is no better, and the judgment of the practitioner is equally shown in knowing when to meddle and when to "stay the obstetric hand," when the soft parts are perfectly relaxed and the dilatation of the os is complete—but not before; if the membranes resist the contractions of the womb they should be ruptured by the attendant. The womb, no longer protected by the amniotic fluid, is excited to increased contraction by contact with the irregularities of the child's body, while its lessened size enables its muscles to act in shorter circuits with increased power. When flexion is taking place this may sometimes be aided and the termination of the labor hastened by pushing the head upward in the absence of pain, so as to free it from the pubes, and then with the finger pressing the occiput forward and downward when the pain returns.

In supporting the perineum it should be gently drawn a little forward. When the perineum is ruptured—and there is no skill or care that will always prevent this accident—the nurse should keep the wound cleansed by proper ablutions and leave it to heal without surgical interference. I have always treated this injury in this manner, and I have never had a ruptured perineum occurring in my own practice that required a surgical operation. In very infrequent cases, the laceration is so extensive as to demand an operation, but if so the operation should be secondary. The patient will be better able to endure it after she has recovered from her confinement, and it may then be made on healthy tissue.

After the child is born I try and get the placenta soon and seldom allow it to remain longer than ten minutes. Its early removal lessens the danger of hæmorrhage ; it puts the patient's mind at rest, and the womb acts better if it is not suffered to remain too long in a state of repose after its contractile efforts on the child have ceased. For this purpose contractions are best secured by manipulations through the abdominal walls, but if the adhesions of the placenta are not soon broken up, or if there is hour-glass contraction, or inertia of the womb, it is better to introduce the hand into the cavity before the os becomes contracted, or the soft parts sore, after the child has passed. During the delivery of the placenta the attendant's hand over the womb should give him constant assurance that the organ preserves its globular form. With this precaution I do not understand how inversion is possible. As a security against post-partum hæmorrhage, the hand should be kept constantly over the womb for about one hour after it is empty, to detect and prevent any tendency in the organ to fall into a state of inertia. Auxiliary to these manipulations, if bleeding is feared, ʒj of the fluid extract of ergot should be administered when the child is nearly expelled or immediately after. Ergot seems to be a hæmostatic, but I have not much confidence in its power to excite uterine contractions, and I think it is best that we should place little reliance on it for this purpose.

When the dilatation of the os and soft parts is complete and the pains are insufficient, I would use forceps rather than suffer too much delay, even in cases that would finally terminate without such interference ; but if the delay is from inertia, care must be taken not to empty the womb until we feel assured that its contractions may be made to follow the slow extraction of the child. The skillful use of forceps is not a source of danger, and when two or three hours of agony can be saved to the mother it is no small consideration. The physician should never use instruments for his own convenience, but only in the interests of the mother or child. If the head can be made to present I would never attempt podalic version. With this presentation the operation of turning is more difficult and dangerous than delivery with the forceps, and I have never attempted it, under such circumstances, without having had occasion to regret it. I cannot adequately recount the praises due the obstetric forceps.

It outranks all else in the armamentarium of surgeon or physician. No other instrument has cut short so much suffering or rescued so many lives. I can easily excuse the anathemas which Dr. Slop poured out on his servant Obadiah, for having tied his in a green-baize bag, with good, hard, honest, devilish tight, hard knots, made *bona fide*, so that the Doctor was unable to reach it in the moment of emergency; anathemas that forced the gentle Uncle Toby to exclaim, "our armies swore terribly in Flanders, but nothing to this—my heart would not let me curse the Devil himself with so much bitterness;" and all this, though the forceps threatened some sore misfortunes to the luckless Tristram, then in the imminent moment of his nativity. The blades, too, have been applied to ornamental, as well as useful, purposes. You will remember that after our city was consumed by fire, many preserved as mementoes of the conflagration various objects found among the debris. Almost the last of these to remain are a lot of the blades of obstetric forceps, which I have annually seen stuck in the earth surrounding a flower bed in private ornamented grounds. I could never quite satisfy myself whether the blades were placed there entirely in the interest of decorative art, or whether the horticulturist did not have a latent belief that they would help to make the flowers come out.

The abdominal bandage after labor, although not indispensable, affords much relief and prevents hæmorrhage. An old linen towel, reaching from the pelvis to the thorax, answers the purpose. After delivery, the bowels are generally paralyzed and constipated, and it used to be the custom to move them in two or three days. Dr. Ingals made it his practice to allow them to remain so for four or five days, and he thought the administration of castor oil was liable to be followed by constipation. He prescribed an enema instead.

As to the diet, it required some attention, the digestive system being generally impaired. He believed potatoes were then specially indigestible. Four or five days after labor, excessive perspiration may take place, and is best relieved by 10 or 12 grains of quinine, though it has nothing to do with malaria. The patient should not use her eyes for reading or needle-work for a month after confinement. A number of patients lose their hair during convalescence. The tr. of cantharides, one part, to five of alcohol, is a good application. No patient should get on her feet before the ninth day.

*Discussion.*—Dr. G. C. Paoli thought it was not always possible to select a good room, and he believed it was criminal on the part of proprietors to rent to poor people basements unfit for any one to live in. As to the use of ergot in labor, he had used it favorably, but the tr. or fl. extr. were not worth much. Half grain doses of ipecac had a special power for contracting the os uteri. He referred prolapse of the womb in many cases to an early recovery from labor, and the parturient woman should not get up till the twelfth day. He regarded the bandage as something indispensable, but a flannel bandage is preferable, on account of perspiration.

Dr. W. T. Montgomery stated that cases of asthenopia are often referable to straining of the eyes after labor. He recommended as a prophylactic against hæmorrhage, to keep the feet and hands of the patient warm.

Dr. Jane E. Walton made it her practice to get the patient up early, that is, not later than the twelfth day; and recommended the use of forceps whenever convenient.

Dr. E. F. Ingals made the query, Does the use of forceps predispose the child to convulsions afterward? He had seen convulsions occur after six months in one case, and after a few weeks in another. He recommended disinfecting injections after labor. They were grateful to the patient. Also, ablutions three times a day.

Dr. E. Ingals, in reference to vaginal infections, said that they should be used whenever something had been retained, and whenever the lochial discharge had an offensive smell. He did not believe that the use of forceps increased in any degree the liability of infants to convulsions. In bad cases of post-partum hæmorrhage he emptied the uterus of all clots and pressed its walls over one hand inside, with the other hand holding the uterus through the parietes of the abdomen. He regarded this as a more efficient compression of the aorta than if pressure was made over the latter. He did not believe that ergot produced contraction of the womb, as generally supposed.

Dr. Tucker agreed with him on that point. H. D. V.

